# White-Light-Emitting Decoding Sensing for Eight Frequently-Used Antibiotics Based on a Lanthanide Metal-Organic Framework

**DOI:** 10.3390/polym11010099

**Published:** 2019-01-09

**Authors:** Mingke Yu, Xu Yao, Xinyu Wang, Yuxin Li, Guangming Li

**Affiliations:** Key Laboratory of Function Inorganic Material Chemistry (MOE), School of Chemistry and Material Science, Heilongjiang University, Harbin 150080, China; m18646858766_2@163.com (M.Y.); iyaoxu@163.com (X.Y.); w5wangxinyu@163.com (X.W.)

**Keywords:** lanthanide, metal-organic framework, white light, antibiotics, multi-selective luminescence sensing, decoding sensing

## Abstract

Developing multi-selective luminescence sensing technology to differentiate serial compounds is very important but challenging. White-light-emitting decoding sensing based on lanthanide metal-organic frameworks (Ln-MOFs) is a promising candidate for multi-selective luminescence sensing application. In this work, three isomorphic Ln-MOFs based on H_3_dcpcpt (3-(3,5-dicarboxylphenyl)-5-(4-carboxylphenl)-1H-1,2,4-triazole) ligand, exhibiting red, blue, and green emission, respectively, have been synthesized by solvothermal reactions. The isostructural mixed Eu/Gd/Tb-dcpcpt is fabricated via the in-situ doping of different Ln^3+^ ions into the host framework, which can emit white light upon the excitation at 320 nm. It is noteworthy that this white-light-emitting complex could serve as a convenient luminescent platform for distinguishing eight frequently-used antibiotics: five through luminescence-color-changing processes (tetracycline hydrochloride, yellow; nitrofurazone, orange; nitrofurantoin, orange; sulfadiazine, blue; carbamazepine, blue) and three through luminescence quenching processes (metronidazole, dimetridazole, and ornidazole). Moreover, a novel method, 3D decoding map, has been proposed to realize multi-selective luminescence sensing applications. This triple-readout map features unique characteristics on luminescence color and mechanism. The mechanism has been systematically interpreted on the basis of the structural analysis, energy transfer and allocation process, and peak fitting analysis for photoluminescence spectra. This approach presents a promising strategy to explore luminescent platforms capable of effectively sensing serial compounds.

## 1. Introduction

Selectivity is a defining characteristic of luminescence sensing, and helpful to recognize even the most similar chemicals [1,2,3,4]. In the past decades, single-selective luminescence sensing for ions (cations and anions), molecules (organic small molecules, gas molecules, and biomacromolecules), and circumstance factors (pH and temperature) have been frequently employed [5,6,7,8,9,10,11,12,13,14,15]. However, investigating the serial identification of complicate and homogeneous compounds requires multi-selective luminescence sensing tools to probe several parameters. Multiplex selectivity provides unprecedented convenience in component recognition of complex systems, contributes to real-time and on-site testing, and is indispensable for deciphering mechanisms of luminescence sensing [16].

Previous research on luminescence sensing primarily focuses on turn-on and -off or luminescence-color-changing sensors [17,18,19,20,21,22,23]. The former utilizes the enhanced or quenching luminescence intensity of monochromatic materials; the latter realizes luminochromatism in terms of multi-emitting materials. However, it is rarely reported to simultaneously consider luminescence color and intensity, which is a potential path toward multi-selective luminescence sensing applications. Accordingly, we hypothesis a novel methodology, called white-light-emitting decoding sensing, to multi-selectively detect serial compounds [24,25,26,27,28]. The perturbation of distinct analytes to each component of white-light-emitting sensors may alter both luminescence color and emission intensity ratios of components, thereby providing an efficient platform to easily and conveniently differentiate each member of the serial compounds. Certainly, to better realize multi-selective luminescence sensing requires that the white-light-emitting sensor features multi-emission of three primary colors (red, green, and blue), non-overlapping peaks, sensitization to invasion, etc.

Based on the above limitations, lanthanide metal-organic frameworks (Ln-MOFs) with white-light emissions are promising candidates for multi-selective luminescence sensing [29,30,31,32]. In addition to the characteristic sharp f-f emission of lanthanide ions, ligand-centered and co-luminescence behaviors in Ln-MOFs frequently emerge through moderating sensitization of ligand to lanthanide ions [33,34]. Therefore, tunable color, and even white light, could be obtained by meticulously tuning the stoichiometric ratio of these emission components [35,36,37,38]. Moreover, each component often presents distinct sensitivity to structural details of the coordination environment, thereby providing a potential multi-selective luminescence sensing platform. Recently, some reports implied the feasibility using white-light-emitting Ln-MOFs as multi-selective luminescence sensing materials. In 2017, the double-selective detection of Ag^+^ and Mn^2+^ ions in water through the luminescence-color-changing process of a white-light-emitting lanthanide coordination complex was reported in our previous work [22]. In 2018, Su et al. successfully achieved luminescence decoding for a series of metal ions in solution and volatile organic compounds in vapor by using Ln-MOFs with red and green-light emission [39]. Although some progress has been made in this regard, the multi-selective luminescence sensing technology based on Ln-MOFs is unreported, meanwhile the cogent system analysis for sensing mechanisms is still a great barrier for the development of this type of sensor.

Alternately, antibiotics as drugs are widely applied in treatment of bacterial infections [40,41]. In recent years, the discharge and abuse of antibiotics led to high levels of antibiotic residues in both surface and groundwater, as well as in drinking water [42]. These antibiotics are highly toxic, carcinogenic, and difficult to biodegrade. Recent investigation shows that the total antibiotics usage in China in 2013 was up to 162,000 tons, resulting in much of the drinking water in China being unsafe, with excessive amounts of antibiotic pollutants [43]. Therefore, detecting these specific pollutants from water is significant. While some luminescent complexes showed a promising potential in single-selectively probing antibiotics, multi-selective platforms for effective and readily available detection of different antibiotics in aqueous solutions have not been well investigated so far, let alone for multi-selective luminescence sensing platforms based on Ln-MOFs.

Bearing this in mind, we designed and synthesized a white-light-emitting material through three isomorphic red-, blue-, or green-light emitting Ln-MOFs based on 3-(3,5-dicarboxylphenyl)-5-(4-carboxylphenl)-1H-1,2,4-triazole (H_3_dcpcpt) rigid ligand. Luminescence sensing for twenty frequently-used antibiotics using this white-light-emitting material was carried out and exhibited good differentiating capability for eight antibiotics on either luminescent color or luminescent intensity. Integrating these excellent features, a 3D decoding map was proposed to exactly distinguish these eight antibiotics. Mechanisms on sensing behavior were also systematically investigated.

## 2. Experimental Section

### 2.1. Materials and Instrumentations

LnCl_3_·6H_2_O was obtained by the reaction of Ln_2_O_3_ and hydrochloric acid. The H_3_dcpcpt (Scheme S1) was purchased from commercial sources (Jinan Henghua Chemical Ltd, Jinan, China) and used with recrystallization. Other chemicals were purchased from HWRK Chem (Beijing, China) and used without purification. All antibiotics were purchased from J&K Scientific Ltd. (Beijing, China).

Fourier Transform Infrared (FT-IR) and Ultraviolet (UV) spectra were performed on PerkinElmer Spectrum 100 spectrophotometer (PerkinElmer, Waltham, MA, USA) and PerkinElmer Lambda 35 spectrometer, respectively. Thermogravimetric analyses (TG-DSC) were recorded in 30–800 °C with 10 °C min^−1^ heating rate by PerkinElmer STA 6000. Powder X-ray diffraction (PXRD) data were conducted on a Rigaku D/Max-3B X-ray diffractometer (Rigaku Corporation, Tokyo, Japan) with Cu Kα as the radiation source (λ = 0.15406 nm) in the angular range 2θ = 5–50° at room temperature. Elemental analyses of C, H, O, and N and inductively coupled plasma atomic emission spectroscopy (ICP-AES) were collected on PerkinElmer 2400 analyzer and HK-2000 spectrometer (PerkinElmer, Waltham, MA, USA), respectively. The photophysical properties were measured by utilizing an Edinburgh FLS 920 fluorescence spectrophotometer and attachments (Edinburgh Instrument, Edinburgh, UK).

### 2.2. Synthesis of Complexes ***1**–**4***

Complexes **1**–**3** were synthesized by solvothermal reactions of 0.27 mmol LnCl_3_·6H_2_O (Ln = Gd **1**, Eu **2**, and Tb **3**), 0.11 mmol H_3_dcpcpt ligand, 8 mL DMF, 2 mL 2.7 M HNO_3_, and 2 mL H_2_O prepared in a Teflon-lined autoclave at 150 °C for 72 h and then slowly reduced to room temperature. The resulting rod-shaped colorless crystals were obtained and washed by DMF and water several times.

For complex **4**, the synthetic methods are the same as the afore-mentioned, except that the lanthanide salt is replaced by a mixed one, according to the molar ratio in Eu:Gd:Tb = 2:2:1.

[Eu(dcpcpt)(H_2_O)]_n_ (**1**). Yield: 0.038 g (66.4 wt%). Anal. Calcd. for C_17_H_9_N_3_O_7_Eu (wt%): C, 39.32; H, 1.75; N, 8.09; O, 21.57. Found (wt%): C, 39.28; H, 1.77; N, 8.03; O, 21.56. IR (KBr pellet, cm^−1^): 3587(s), 1677(s), 1585(s), 1428(m), 1361(s), 780(m). UV-vis (MeOH, nm): 204.

[Gd(dcpcpt)(H_2_O)]_n_ (**2**). Yield: 0.032 g (55.4 wt%). Anal. Calcd. for C_17_H_9_N_3_O_7_Gd (wt%): C, 38.93; H, 1.73; N, 8.01; O, 21.35. Found (wt%): C, 39.02; H, 1.77; N, 7.98; O, 21.57. IR (KBr pellet, cm^−1^): 3579(s), 1673(s), 1594(s), 1437(m), 1361(s), 785(m). UV-vis (MeOH, nm): 204.

[Tb(dcpcpt)(H_2_O)]_n_ (**3**). Yield: 0.035 g (60.3 wt%). Anal. Calcd. for C_17_H_9_N_3_O_7_Tb (wt%): C, 38.80; H, 1.72; N, 7.99; O, 21.28. Found (wt%): C, 38.88; H, 1.75; N, 7.91; O, 21.17. IR (KBr pellet, cm^−1^): 3586(s), 1669(s), 1585(s), 1437(m), 1353(s), 789(m). UV-vis (MeOH, nm): 204.

[Eu_0.41_Gd_0.42_Tb_0.17_(dcpcpt)(H_2_O)]_n_ (**4**). Yield: 0.030 g (52.6 wt%). Anal. Calcd. for C_17_H_9_N_3_O_7_Ln (wt%): C, 39.07; H, 1.74; N, 8.04; O, 21.43. Found (wt%): C, 39.02; H, 1.75; N, 8.00; O, 21.37. IR (KBr pellet, cm^−1^): 3554(s), 1677(s), 1594(s), 1428(m), 1361(s), 788(m). UV-vis (MeOH, nm): 204.

### 2.3. Luminescence Sensing Experiments

To obtain a stable suspension, the crystal samples of complex **4** were finely ground and well-dispersed in water, forming a 0.01 M suspension by ultrasonication treatment for 30 min. Then the suspension was applied for luminescence sensing analyses at room temperature. For the sensing experiments, 1 mL different antibiotic aqueous solutions with a concentration of 10^−4^ M were added to the suspension with the same quantity to examine the potential of complex **4** to selectively recognize antibiotics. The quenching constant (K_SV_) for every antibiotic was calculated, using the Stern-Volmer (SV) equation: I_0_/I = 1 + K_SV_[C]. The I_0_ and I represent the lumines-cent intensities in the absence and presence of antibiotics, and [C] is the concentration of antibiotics. In addition, the limitation of detection (LOD = 3σ/K_SV_) of the sensor for antibiotics is calculated in term of the ratio of K_SV_ and standard deviation for three times replicating fluorescence measurements of blank solutions (σ = 0.012).

### 2.4. X-ray Crystallographic Analysis

Crystal data for complex **3** were collected on an Oxford Xcalibur Gemini Ultra diffractometer (Oxford Instrument, Oxford, UK), using graphite-monochromated Mo Kα radiation (λ = 0.71073 Å) at room temperature. The structure of complex **3** was solved by using Patterson methods (SHELXS-97), expanded using Fourier methods, and refined using SHELXL-2016 (full-matrix least-squares on F2) and Olex2 programs packages (v1.2, Durham University, Durham, UK, 2004–2019). All non-hydrogen atoms were refined anisotropically. Empirical absorption corrections based on equivalent reflections were applied. The resulting new files were applied to further refine the structures.

## 3. Results and Discussions

### 3.1. Structure Analysis of Complexes ***1**–**3***

**Figure 1:**
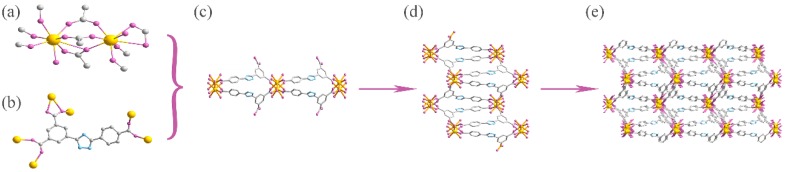
(**a**) Coordination environment of central Tb^3+^ ions in **3** with hydrogen atoms for clarity. (**b**) Coordination mode of dcpcpt^3−^ ligand. (**c**) 1D chain. (**d**) 2D network. (**e**) 3D framework.

A solvothermal synthesis of H_3_dcpcpt with LnCl_3_∙6H_2_O yields three Ln-MOFs (Ln(dcpcpt)(H_2_O))_n_ (Ln = Eu **1**, Gd **2**, Tb **3**). Powder X-ray diffraction analysis confirms the isomorphism of complexes **1**–**3** (Figure S2). Systematic analysis on the structure of complex **3** was subsequently investigated. Firstly, the structure could be clearly observed through the X-ray diffraction analysis, though the complete crystal structures of the complexes could not be obtained after several attempts under room and low temperature. In the typical structure of **3**, the coordination polymer features a 3D framework structure crystalizing in the monoclinic P121/c1 space group. Each Tb^3+^ ion is eight-coordinated by seven oxygen atoms from six deprotonated dcpcpt^3-^ ligands and one oxygen atom from water molecules, forming the bicapped trigonal prism geometry (Figure S1). Meanwhile, the neighboring Tb^3+^ ions are linked by carboxylate oxygen atoms from the ligand, further forming the binuclear (Tb_2_(COO)_6_)_n_ secondary building unit (SBU) (Figure 1a). The adjacent units are linked by ligand to form 1D chains in k_1_-k_1_-μ_2_ mode, which are further bridged by dcpcpt^3-^ to form a 2D network in k_1_-k_2_-μ_2_ mode (Figure 1c,d). The adjacent 2D networks ultimately accumulate into a 3D frame-work (Figure 1e). Two open 1D windows, with a size of about 16.7 × 5.47 Å and 15.43 × 4.13 Å, are represented. In the crystal structure, the ligands remain nearly planar, exhibiting good rigidity. From the perspective of topology, the framework can be viewed as a (3,6)-connected 3D topology due to both the dcpcpt^3-^ bridge three carboxylate groups and that the binuclear [Tb_2_(COO)_6_]_n_ SBUs being connected by six carboxylate groups.

Secondly, FT-IR and TG-DSC data further confirm the structures of complexes **1**–**3** (Figures S3 and S4). In the typical FT-IR spectra of complex **3**, the broad band around 3586 cm^−1^ is attributed to the characteristic peak of the O–H bond, suggesting the existence of water molecules in the complexes (Figure S3). The asymmetrical and symmetrical stretching vibrations of carboxylate groups are observed around 1669 and 1437 cm^−1^. Both the presence of the 1669 cm^−1^ peak and the absence of the 1713 cm^−1^ peak indicate the successful deprotonation of the carboxylate groups and coordination to the lanthanide ions. TG-DSC analysis shows the presence of the coordinated molecules in complexes (Figure S4). In the typical complex **3** spectrum, a rapid weight loss of 64.7% in the temperature range of 360–500 °C is attributed to the dcpcpt ligand. The remaining 28.8% of the weight belongs to the Eu_2_O_3_. The ratio of 64.7%:28.8% implies that the ratio of dcpcpt^3−^ (molar molecular mass: 350.04) and Eu^3+^ (molar molecular mass of Eu_2_O_3_: 351.83) in complex **3** is nearly 1:1. Moreover, the gradual weight loss of 4.9% in temperature ranging from 130 to 250 °C proposed the existence of one coordinated water molecule. According to the above FT-IR and thermal analysis, together with elemental analysis data, the molecule formula of [Ln(dcpcpt)(H_2_O)]_n_ is further confirmed. Similar patterns of FT-IR, TG-DSC, UV-vis, and PXRD confirm the isomorphism of complexes **1**–**3** (Figures S2–S5).

### 3.2. Luminescent Properties of Complexes ***1**–**3***

**Figure 2:**
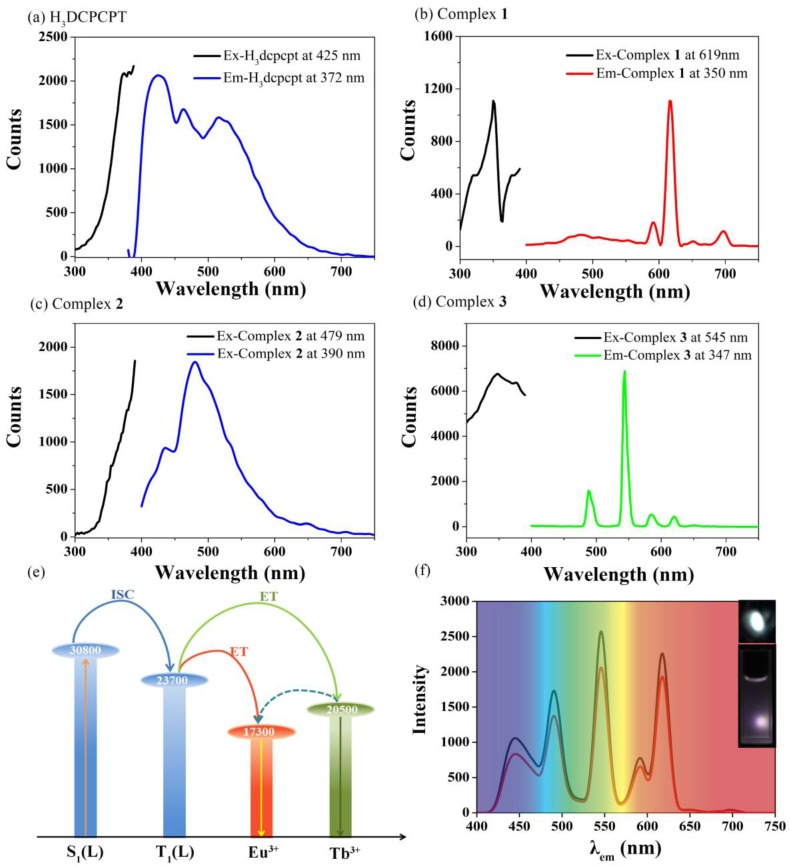
Solid-state photoluminescent spectra of (**a**) H_3_dcpcpt, (**b**) **1**, (**c**) **2**, and (**d**) **3**. (**e**) Simplified schematic diagram of energy transfer process. (**f**) Luminescent spectra and optical photographs of complex **4** in solid state and well-dispersed 0.01 M aqueous solution under the excitation of 320 nm.

The solid state photophysical properties of H_3_dcpcpt ligand and complexes **1**–**3** have been investigated (Figure 2a,d). As shown in Figure 2a, H_3_dcpcpt ligand possesses gradually increasing absorption at 300–390 nm (Figure 2a). Upon the excitation at 372 nm, H_3_dcpcpt ligand presents a blue-green emission from 420 to 550 nm. Sensitized by light-harvesting organic linkers, complex **1** exhibits the strongest absorption at 350 nm by monitoring the strongest emission at 614 nm of the Eu^3+^ ion (Figure 2b). The emission of complex **1** under excitation at 350 nm displays co-luminescence of weak ligand luminescence around 485 nm and sharp characteristic peaks of the Eu^3+^ ion at 591, 614, 651, and 699 nm, which are attributed to ^5^D_0_ → ^7^F*_J_* (*J* = 1, 2, 3, and 4) transitions, respectively. The intense red luminescence of the Eu^3+^ ion is rendered by the stronger electric dipole transition (^5^D_0_ → ^7^F_2_) compared to the magnetic dipole transition (^5^D_0_ → ^7^F_1_) [44]. The unsplit band of the ^5^D_0_ → ^7^F_1_ transition suggests a highly symmetric coordination environment of the Eu^3+^ ion [45]. For complex **2**, it presents a similar absorption band comparing with ligand, yet distinct emission bands around 480 nm (Figure 2c). Complex **3** displays the sharp characteristic peaks of the Tb^3+^ ion at 491, 545, 584, and 619 nm, corresponding to the ^5^D_4_ → ^7^F_J_ (J = 6, 5, 4 and 3) transitions, respectively (Figure 2d). The characteristics of luminescence emission in complexes **2** and **3** indicate that the antenna effect occurs; that is, energy migration takes place upon ligand absorption, followed by intersystem crossing S1 → T1 and antenna T1 → f transfer, and then generating f–f emissions of Eu^3+^ and Tb^3+^ ions (Figure 2e). However, both of the facts of co-luminescence of complex **2** and stronger luminescence intensity of complex **3** suggests that H_3_dcpcpt is more suitable for sensitizing the Tb^3+^ ion.

### 3.3. White-Light Emission of Complex ***4***

Based on the isomorphism of complexes **1**–**3** exhibiting three primary luminescent colors, it would be probable to construct white-light-emitting materials by meticulously tuning the stoichiometric ratio of Eu^3+^, Gd^3+^, and Tb^3+^ ions. As a result, the corresponding three-component Ln-MOF, [Eu_0.41_Gd_0.42_Tb_0.17_(dcpcpt)(H_2_O)]_n_ (**4**), is in-situ synthesized, with a 2:2:1 feed ratio of Eu^3+^:Gd^3+^:Tb^3+^ ions. The accurate molar ratio of 41:42:17 is yielded by inductively coupled plasma (ICP) analysis (Table S1). PXRD, FT-IR, UV-vis, and TG-DSC reveal that complex **4** is isomorphic with **1**–**3** (Figures S1 and S3–S5). According to Figure 2f, a white-light emission, with CIE coordinates of (0.330, 0.338), which is close to those of pure white light (0.333, 0.333), emerges upon excitation at 320 nm. In addition, the luminescence property of complex **4** remains intact upon dissolving to form a well-dispersed 0.01 M aqueous suspension.

Luminescence life-time data of complex **4** implies the existence of energy transfer from Tb^3+^ to Eu^3+^ ion (Table S2). To quantify the efficiency of this inter-metallic transfer (η_ET_), the relationship (η_ET_ = 1 − τ_obs_/τ_0_) has been used, where τ_obs_ and τ_0_ are the observed luminescent lifetimes of Tb^3+^ in presence and in absence of an acceptor Eu^3+^ ion [46]. According to Table S2, the calculated inter-metallic energy transfer efficiency in this white-light-emitting material is approximately 77.4%. Therefore, complex **4** evidences a very efficient Tb^3+^-to-Eu^3+^ energy transfer that compares well with what was observed in previously reported systems, for which η_ET_ was about 90% [47]. This is probably related to the anisotropic character of the metallic distribution in this crystal structure.

### 3.4. Luminescence Sensing toward Antibiotics

**Figure 3:**
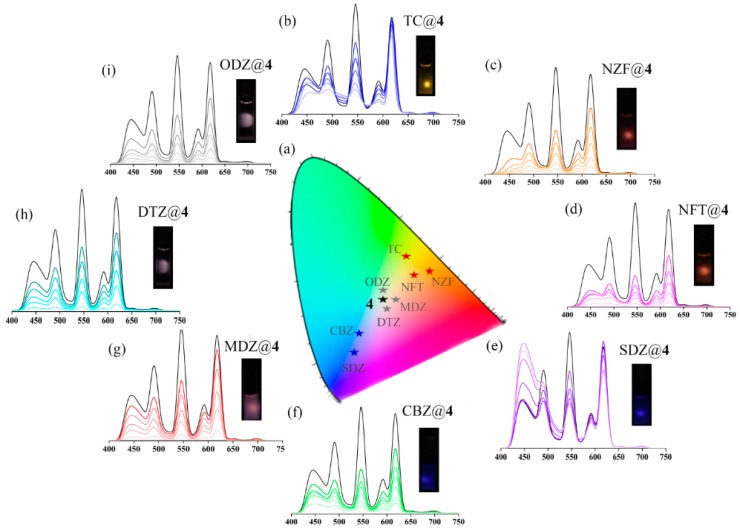
(**a**) CIE chromaticity diagram showing the luminescence color of the sensor with various antibiotics of 10^−4^ M aqueous solution. Con-centration-dependent luminescence spectra of the sensor with distinct concentrations of (**b**) tetracycline hydrochloride (TC), (**c**) nitrofurazone (NZF), (**d**) nitrofurantoin (NFT), (**e**) sulfadiazine (SDZ), (**f**) carbamazepine (CBZ), (**g**) metronidazole (MDZ), (**h**) dimetridazole (DTZ), and (**i**) ornidazole (ODZ). Insets are optical photographs of the antibiotic@**4** with various antibiotics of 10^−4^ M aqueous solution.

To explore the capability of this white-light-emitting Ln-MOF to differentiate antibiotics, various antibiotics aqueous solutions with the concentration of 10^−4^ M (analytes) were added into the well-dispersed 0.01 M aqueous suspension of complex **4** (sensor) to form antibiotic@**4**. Twenty frequently-used antibiotics in six classes were selected and checked, including β-lactams (penicillin, PCL; amoxicillin ACL; cefixime CFX; cephradine CFD), aminoglycosides (gentamicin GTM; kanamycin KNM), macrolides (roxithromycin ROX; azithromycin AZM), quinolones (ciprofloxacin CPFX; norfloxacin NFX), nitrofurans (nitrofurazone NZF; nitrofurantoin NFT), nitroimidazole (metronidazole MDZ; dimetridazole DTZ; ornidazole ODZ), and others (tetracycline hydrochloride TC; vancomycin VCC; sulfadiazine SDZ; lincomycin LCC; carbamazepine CBZ) (Figure S6). Interestingly and notably, the luminescence color and intensity feature heavy dependence on the categories of antibiotics (Figure 3a). After introducing TC and nitrofurans (NZF and NFT), the luminescence of complex **4** turns to yellow and orange, respectively. For SDZ and CBZ, the sensor turns to blue. In addition, the sensor exhibits obvious luminescent quenching during the detection of nitroimidazole antibiotics such as MDZ, DTZ, and ODZ. According to the result, this white-light-emitting material based on Ln-MOFs could realize multi-selective detection for eight frequently-used antibiotics in aqueous solution, which, to our knowledge, is rarely reported. This contribution results from a series of factors: (1) the simultaneous consideration of luminescence color and intensity, which increases the selectivity; and (2) the excellent sensitivity of white-light-emitting Ln-MOFs. Subsequently, quantitative luminescence titration experiments of white-light-emitting sensors to every antibiotic are carried out to explore the luminescence-color-changing and quenching processes.

As the concentration of TC increases, the intensity of the ligand component (400–500 nm) and Tb^3+^ (545 nm) component rapidly decreases, meanwhile the Eu^3+^ (614 nm) component nearly remains unchanged, which contributes to the luminescence color changes from white to yellow (Figure 3b). In addition, the ligand component of **4** after the introduction of TC possesses obvious bathochromic shifts from the blue to green area, demonstrating the decrease in the energy level of the π* orbits of the ligand [22]. According to the inset image of Figure 3b, the TC@**4** with the TC concentration of 10^−4^ M presents obvious yellow emissions upon excitation at 320 nm, demonstrating the visual LOD of the sensor to TC is up to the ppm level. The Stern-Volmer plot of TC@**4** based on the intensity at 545 nm illustrates good linear correlation, and the value of K_SV_ is estimated to be 4.06 × 10^4^ M^−1^, revealing a strong sensing performance on the luminescence of complex **4** (Figure S7). The LOD of this white-light-emitting material to TC is 887 ppb, meaning that the present sensor possesses highly sensitive properties for TC in aqueous solution.

For nitrofurans, including NZF and NFT, all the emissions of ligand, Tb^3+^, and Eu^3+^ components decreased rapidly. Ligand component has obvious bathochromic shifts, like TC@**4** (Figure 3c,d). The quenching rate of Tb^3+^ component was more rapid than that of Eu^3+^ component. Based on the abovementioned factors, NZF@**4** and NFT@**4**, with the concentration of 10^−4^ M, present obvious orange emissions, according to the inset image of Figure 3c,d. The values of K_SV_ for NZF@**4** and NFT@**4**, based on the luminescence intensity at 545 nm, are 1.03 × 10^4^ M^−1^ and 1.90 × 10^5^ M^−1^, respectively, and LODs are 3.50 ppm and 189 ppb, respectively (Figure S7).

Despite the blue emission, variation trends for each component were different in SDZ@**4** and CBZ@**4**. For SDZ, the intensity of Eu^3+^ component remained, Tb^3+^ component decreased, and the ligand component increased. For CBZ, all the ligand, Tb^3+^, and Eu^3+^ components decreased rapidly. The values of K_SV_ for SDZ@**4** and CBZ@**4**, based on the luminescence intensity at 545 nm, were 1.90 × 10^4^ M^−1^ and 9.64 × 10^4^ M^−1^, respectively, and LODs were 1.89 ppm and 373 ppb, respectively (Figure S7).

For nitroimidazole antibiotics (MDZ@**4**, DTZ@**4** and ODZ@**4**), the intensity of each luminescence component rapidly decreased with a nearly equal proportion, thus, white-light emission remained after antibiotic sensing. The values of K_SV_ for MDZ@**4** based on ligand, Eu^3+^, and Tb^3+^ components were estimated as 1.37 × 10^5^ M^−1^, 3.58 × 10^4^ M^−1^, and 1.66 × 10^5^ M^−1^, respectively, and LODs were 263 ppb, 1.01 ppm, and 217 ppb (Figure S8). For DTZ@**4**, K_SV_ were 1.19 × 10^5^ M^−1^, 8.93 × 10^4^ M^−1^, and 1.64 × 10^5^ M^−1^, respectively, and LODs were 303 ppb, 403 ppb, and 219 ppb. For ODZ@**4**, K_SV_ were 2.00 × 10^5^ M^−1^, 1.42 × 10^5^ M^−1^, and 2.54 × 10^5^ M^−1^, respectively, and LODs were 180 ppb, 250 ppb, and 142 ppb. To the best of our knowledge, the LODs in this work were far superior to most of the luminescent MOFs for detecting nitroimidazole antibiotics in aqueous solutions.

According to the abovementioned results, the as-synthesized Ln-MOF with white-light emission showed excellent sensing ability for frequently-used antibiotics, both on selectivity and sensitivity. The eight antibiotics@**4** hold the minimal LOD order of NZF > SDZ > TC > CBZ > DTZ > MDZ > NFT > ODZ. According to Table 1, the K_SV_ and LODs of antibiotics@**4** in this contribution are far superior to most reported fluorescence probes for these antibiotics [42,48,49,50,51,52,53,54], and meets the standard of U.S. Environmental Protection Agency and World Health Organization for the maximum allowable level of antibiotics in drinking water (Table 1) [5,55]. Additionally, we find that complex **4** can be regenerated and reused for a significant number of cycles by centrifugation of the solution after use and washing several times with water. In addition, complex **4** could realize successful sensing within 5 min, illustrating fast sensing ability.

### 3.5. Sensing Mechanism toward Antibiotics

**Scheme 1:**
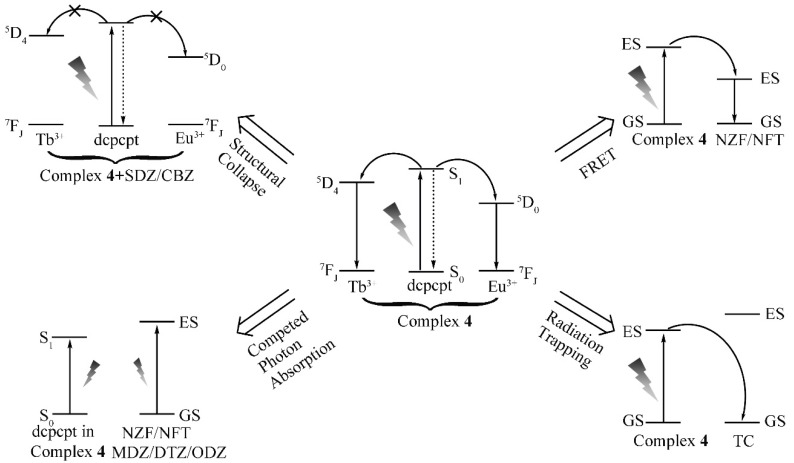
Effect of antibiotics on the antenna effect of complex **4**.

**Figure 4:**
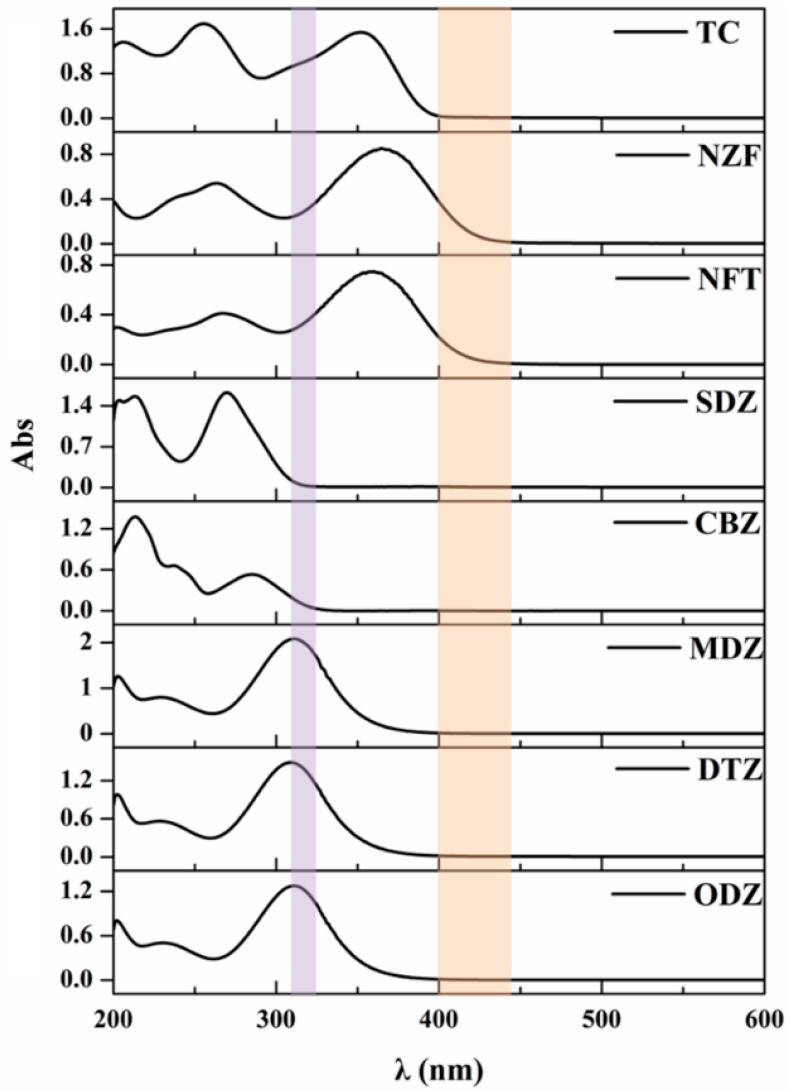
UV-vis spectra of eight frequently-used antibiotics.

The underlying mechanism of antibiotic sensing of complex **4** is investigated according to the experiments. The FT-IR patterns of complex **4** are investigated before and after luminescent sensing for each antibiotic (Figure S9). Except for SDZ@**4** and CBZ@**4**, other antibiotics@**4** hold undiscernible FT-IR patterns, meaning that the alternation on luminescence color and intensity does not result from the breakdown of the host framework for TC@**4**, NZF@**4**, NFT@**4**, MDZ@**4**, DTZ@**4**, and ODZ@**4**. For SDZ@**4** and CBZ@**4**, PXRD patterns implied structural alternation after introducing antibiotics, which may originate from the strong interaction of antibiotic molecules to ligand molecules or lanthanide ions (Figure S10). The structural alternation attenuates antenna effects and increases the luminescence intensity ratio of ligand to lanthanide ions, rendering the luminescence-color-changing process to blue (Scheme 1). Moreover, CBZ possesses weak absorption at 320 nm, according to the UV-vis spectra of antibiotics, so CBZ@**4** also has a quenching effect due to competed photon absorption, along with the luminescence-color-changing process (Figure 4).

Further studies of the energy transfer and allocation between antibiotics and frameworks were carried out. UV-vis absorption spectra of NZF and NFT in aqueous solution showed the greatest spectral overlap at 400–450 nm, with the emission of white-light-emitting Ln-MOF (Figure 2f and Figure 4). This caused the occurence of fluorescence resonance energy transfer (FRET) because the light emitted by Ln-MOF could be absorbed by NZF and NFT molecules [56]. Therefore, NZF@**4** and NFT@**4** presented a luminescence-color-changing process to orange with the decrease of blue light. Like CBZ@**4**, these two antibiotics also had weak absorption at 320 nm, so they also showed competing photon absorption along with the abovementioned luminescence-color-changing process (Figure 4). According to the UV-vis spectra at 320 nm, MDZ, DTZ, and ODZ presented strong absorption, thereby providing competing photon absorption between antibiotics and frameworks [57]. Accordingly, MDZ@**4**, DTZ@**4**, and ODZ@**4** showed obvious quenching effects.

**Figure 5:**
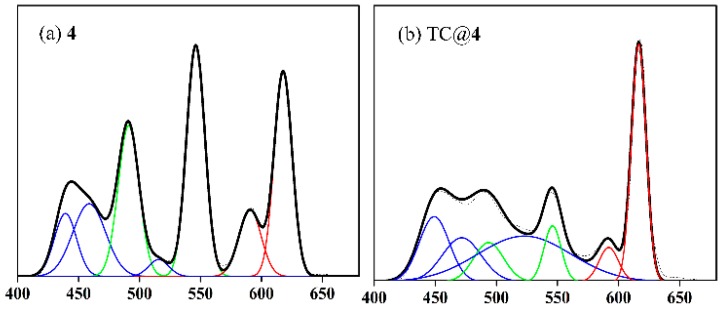
Fitting peaks analysis for complex (**a**) **4** and (**b**) TC@**4**.

Comparatively and interestingly, TC@**4** featured unique photoluminescence properties. To better understand the mechanism of luminescence sensing to antibiotics, fitting peaks technology to luminescence peaks was applied (Figure 5 and Tables S3 and S4). As shown in Figure 5a, the luminescence spectrum of white-light-emitting complex **4** could be fitted into three components: ligand component at 439, 459, and 516 nm; Tb^3+^ component at 490 and 545 nm; Eu^3+^ component at 590 and 614 nm. For yellow emission of TC@**4** in Figure 5b, the ligand component possessed an obvious bathochromic shift, suggesting the existence of interaction between Ln-MOF and an invader, such as π-π interactions and hydrogen bonds [22]. The bathochromic shift of ligand luminescence peaks suggests the existence of radiation trapping between complex 4 and TC [58,59,60].

### 3.6. 3D Decoding Map

**Figure 6:**
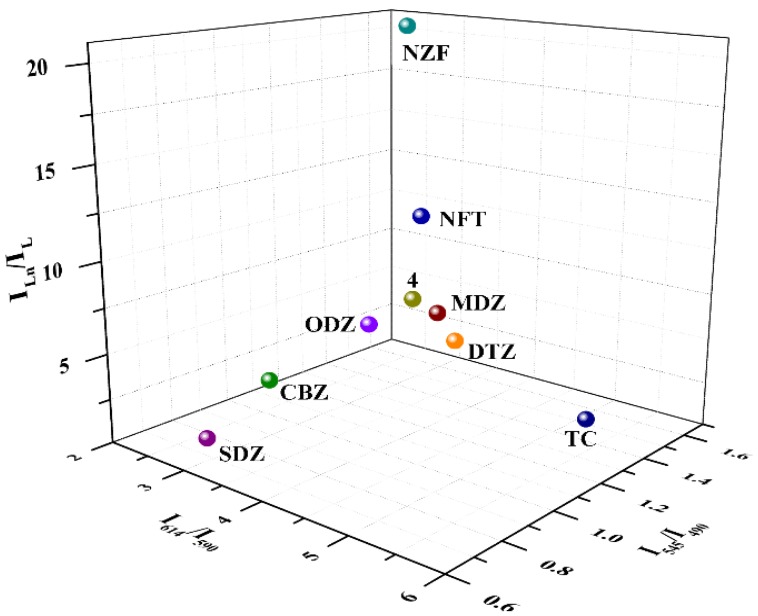
3D decoding maps of antibiotics based on the emission intensity ratio I_614_/I_590_, I_545_/I_490_, I_Ln_/I_L_ as x-, y-, and z-axis, respectively.

According to the abovementioned analysis, each antibiotic can characteristically tune the energy transfer efficiency of ligand-to-metal energy transfer or modulate the energy allocation between analytes and the sensor [39]. Based on these interesting features, a reasonable 3D decoded map for different antibiotics can be fabricated by utilizing I_614_/I_590_, I_545_/I_490_, and I_Ln_ or I_L_ (I_Ln_ equals to the sum of intensities at 617 and 545 nm; I_L_ equals to the maximum intensity ranged from 425 to 475 nm) as the x-, y-, and z-axis, respectively. As shown in Figure 6, eight antibiotics@**4** could be recognized and differentiated in this 3D decoding map, realizing multi-selective luminescence sensing applications.

A series of phenomena and rules could be further observed and extrapolated from the 3D decoding map for the white-light-emitting Ln-MOFs. On the luminescence color, the increase of x-values implied the ascending proportion of green light; the increase of y-value meant the descending proportion of blue light; the increase of z-value suggested the ascending proportion of yellow light. On the sensing mechanism, the enhanced sensitization to Eu^3+^ ion from ligand or Tb^3+^ ion showed greater x-value; the structural collapse implied deviation from the y-value of white-light-emitting Ln-MOFs; the weaker antenna effect illustrated greater z-value.

Given that escalating white-light-emitting Ln-MOFs are applied as sensors, the multi-selective luminescence sensing method via present tri-readout cubic identification scheme was expected to be more promising. This method was reliable, convenient, accurate, and powerful. Comparing the traditional luminescence-color-changing and turn-on and -off sensing, this multi-selective luminescence sensing method possesses outstanding multi-selective luminescence sensing ability, and is of practical significance.

## 4. Conclusions

Solvothermal reactions of H_3_dcpcpt and LnCl_3_∙6H_2_O yield three isomorphic 3D lanthanide metal-organic frameworks emitting red, blue, and green light, respectively. A white-light-emitting material could be fabricated by tuning the stoichiometric ratio of three Ln-MOFs. A novel sensing process called multi-selective luminescence sensing with high sensitivity was designed and developed on the basis of simultaneous consideration of luminescence color and intensity, which could be effectively applied to multi-selective luminescence sensing toward eight frequently-used antibiotics. To the best of our knowledge, it is the first example of white-light-emitting Ln-MOFs to detect serial homogeneity. Moreover, structural analysis, energy transfer and allocation process, and peak fitting analysis for photoluminescence spectra are systematically utilized to interpret the underlying mechanism after antibiotic invasion. Notably and innovatively, a 3D decoding map is proposed to differentiate these antibiotics and implicitly reveal the sensing mechanism. The abundant details on luminescence color, intensity, and sensing mechanism in the map substantiate that this approach provides potentially practical luminescence sensing for multi-selective detection.

## Figures and Tables

**Table 1:** Comparison of luminescent materials for sensing antibiotics.

Materials	Color	Approach	Objectives	Limitation of Detection (ppm)	Ref.
Zr-ettc	Green	quenching	TC	0.030	[48]
Cd-tbaed	Blue	quenching	NZF	0.162	[42]
Cd-tbaed	Blue	quenching	NTF	0.274	[42]
AMOC	Blue	quenching	SDZ	0.480	[49]
CDs	Blue	quenching	CBZ	1.930	[50]
CNDs	Blue	quenching	MDZ	0.279	[51]
Eu^3+^/Al^3+^-bta	red	quenching	DTZ	0.377	[52]
Eu-tatab	red	quenching	ODZ	0.800	[53]
Eu/Gd/Tb-dcpcpt	White	multi-selective luminescence sensing	TC	0.887	This work
			NZF	2.770	
			NFT	0.189	
			SDZ	1.890	
			CBZ	0.373	
			MDZ	0.217	
			DTZ	0.219	
			ODZ	0.142	

## Supplementary Materials

The following are available online at http://www.mdpi.com/2073-4360/11/1/99/s1, Scheme S1: The structure of the ligand H3dcpcpt. Figure S1: PXRD patterns of complex **3** simulated from the X-ray single-crystal structure and as-synthesized samples of complexes **1**–**4**, Figure S2: (**a**) The asymmetric unit of complex **3**. All hydrogen atoms and solvent molecules are omitted. (**b**) Coordination polyhedrons of Tb^3+^ ion, Figure S3: FT-IR spectra of ligand and complexes **1**–**4**, Figure S4: TG curves of complexes **1**–**4**, Figure S5: UV-vis spectra of ligand and complexes **1**–**4**, Figure S6: Emission spectra of antibiotics@**4** excited at 320 nm, Figure S7: Stern-Volmer plot of TC@**4**, NZF@**4**, NFT@**4**, SDZ@**4** and CBZ@**4**antibiotics@**4**, Figure S8: Stern-Volmer plot of MDZ@**4**, DTZ@**4** and ODZ@**4**, Figure S9: FT-IR patterns of complex **4** before and after sensing antibiotics, Figure S10: PXRD patterns of complex **4** before and after sensing SDZ and CBZ. Table S1: Elemental analysis of lanthanide ions by ICP for complex **4**, Table S2: Luminescence lifetime of complexes **1**–**4**, Table S3: Peak analysis for **4**, Table S4: Peak analysis for TC@**4**.

## References

[1] Huang R., Wei Y., Dong X., Wu X., Du C., Zang S., Mark T. (2017). Hypersensitive dual-function luminescence switching of a silver-chalcogenolate cluster-based metal–organic framework. Nat. Chem..

[2] Gong T., Li P., Sui Q., Chen J., Xu J., Gao E. (2018). A stable electron-deficient metal–organic framework for colorimetric and luminescence sensing of phenols and anilines. J. Mater. Chem. A.

[3] Liu C., Zhang R., Lin C., Zhou L., Cai L., Kong J., Yang S., Han K., Sun Q. (2017). Intraligand Charge Transfer Sensitization on Self-Assembled Europium Tetrahedral Cage Leads to Dual-Selective Luminescent Sensing toward Anion and Cation. J. Am. Chem. Soc..

[4] Wu S., Lin Y., Liu J., Shi W., Yang G., Cheng P. (2018). Rapid Detection of the Biomarkers for Carcinoid Tumors by a Water Stable Luminescent Lanthanide Metal–Organic Framework Sensor. Adv. Funct. Mater..

[5] Khandelwal P., Singh D., Sadhu S., Poddar P. (2015). Study of the nucleation and growth of antibiotic labeled Au NPs and blue luminescent Au_8_ quantum clusters for Hg^2+^ ion sensing, cellular imaging and antibacterial applications. Nanoscale.

[6] Lu T., Zhang L., Sun M., Deng D., Su Y., Lv Y. (2016). Amino-Functionalized Metal-Organic Frameworks Nanoplates-Based Energy Transfer Probe for Highly Selective Fluorescence Detection of Free Chlorine. Anal. Chem..

[7] Yang Y., Feng Y., Qiu F., Iqbal K., Wang Y., Song X., Wang Y., Zhang G., Liu W. (2018). Dual-Site and Dual-Excitation Fluorescent Probe That Can Be Tuned for Discriminative Detection of Cysteine, Homocystein, and Thiophenols. Anal. Chem..

[8] Xu X., Yan B. (2016). Eu(III) functionalized Zr-based metal-organic framework as excellentfluorescent probe for Cd^2+^detection in aqueous environment. Sens. Actuators B Chem..

[9] Wang L., Fan G., Xu X., Chen D., Wang L., Shi W., Cheng P. (2017). Detection of polychlorinated benzenes (persistent organic pollutants) by a luminescent sensor based on a lanthanide metal–organic framework. J. Mater. Chem. A.

[10] Hao J., Yan B. (2017). Determination of Urinary 1-Hydroxypyrene for Biomonitoring of Human Exposure to Polycyclic Aromatic Hydrocarbons Carcinogens by a Lanthanide-functionalized Metal-Organic Framework Sensor. Adv. Funct. Mater..

[11] Chen B., Xiang S., Qian G. (2010). Metal-Organic Frameworks with Functional Pores for Recognition of Small Molecules. Acc. Chem. Res..

[12] Hao J., Yan B. (2016). Simultaneous determination of indoor ammonia pollution and its biological metabolite in the human body with a recyclable nanocrystalline lanthanide-functionalized MOF. Nanoscale.

[13] Xu X., Yan B. (2017). Intelligent Molecular Searcher from Logic Computing Network Based on Eu(III) Functionalized UMOFs for Environmental Monitoring. Adv. Funct. Mater..

[14] Xia T., Cui Y., Yang Y., Qian G. (2017). Highly Stable Mixed-Lanthanide Metal–Organic Frameworks for Self-Referencing and Colorimetric Luminescent pH Sensing. ChemNanoMat.

[15] Lu Y., Yan B. (2014). A ratiometric fluorescent pH sensor based on nanoscale metal–organic frameworks (MOFs) modified by europium(III) complexes. Chem. Commun..

[16] Kovacs D., Lu X., Mészáros L., Ott M., Andres J., Borbas K. (2017). Photophysics of Coumarin and Carbostyril-Sensitized Luminescent Lanthanide Complexes: Implications for Complex Design in Multiplex Detection. J. Am. Chem. Soc..

[17] Cui Y., Zhang J., He H., Qian G. (2018). Photonic functional metal–organic frameworks. Chem. Soc. Rev..

[18] Luan K., Meng R., Shan C., Cao J., Jia J., Liu W., Tang Y. (2018). Terbium Functionalized Micelle Nanoprobe for Ratiometric Fluorescence Detection of Anthrax Spore Biomarker. Anal. Chem..

[19] Dong Y., Cai J., Fang Q., You X., Chi Y. (2016). Dual-Emission of Lanthanide Metal−Organic Frameworks Encapsulating Carbon-Based Dots for Ratiometric Detection of Water in Organic Solvents. Anal. Chem..

[20] Lustig W., Mukherjee S., Rudd N., Desai A., Li J., Ghosh S. (2017). Metal–organic frameworks: functional luminescent and photonic materials for sensing applications. Chem. Soc. Rev..

[21] Guo Y., Feng X., Han T., Wang S., Lin Z., Dong Y., Wang B. (2014). Tuning the Luminescence of Metal−Organic Frameworks for Detection of Energetic Heterocyclic Compounds. J. Am. Chem. Soc..

[22] Li Y., Li S., Yan P., Wang X., Yao X., An G., Li G. (2017). Luminescence-colour-changing sensing of Mn^2+^ and Ag^+^ ions based on a white-light-emitting lanthanide coordination polymer. Chem. Commun..

[23] Shi J., Deng Q., Li Y., Zheng M., Chai Z., Wan C., Zheng Z., Li L., Huang F., Tang B. (2018). A Rapid and Ultrasensitive Tetraphenylethylene-Based Probe with Aggregation-Induced Emission for Direct Detection of α-Amylase in Human Body Fluids. Anal. Chem..

[24] Wang M., Guo G. (2016). Inorganic–organic hybrid white light phosphors. Chem. Commun..

[25] Kumar K., Chorazy S., Nakabayashi K., Sato H., Sieklucka B., Ohkoshi S. (2018). TbCo and Tb_0.5_Dy_0.5_Co layered cyanido-bridged frameworks for construction of colorimetric and ratiometric luminescent thermometers. J. Mater. Chem. C.

[26] Zhao S., Zhang H., Wang L., Chen L., Xie Z. (2018). Facile preparation of a tetraphenylethylene-doped metal–organic framework for white light-emitting diodes. J. Mater. Chem. C.

[27] Yang D., Tian Y., Cao X., Zheng S., Ju Q., Huang W., Fang Z. (2017). A Series of Lanthanide-Based Metal−Organic Frameworks: Synthesis, Structures, and Multicolor Tuning of Single Component. Inorg. Chem..

[28] Peedikakkal A., Quah H., Chia S., Jalilov A., Shaikh A., Al-Mohsin H., Yadava K., Ji W., Vittal J. (2018). Near-White Light Emission from Lead(II) Metal−Organic Frameworks. Inorg. Chem..

[29] Meyer L., Schönfeld F., Müller-Buschbaum K. (2014). Lanthanide based tuning of luminescence in MOFs and dense frameworks – from mono- and multimetal systems to sensors and films. Chem. Commun..

[30] Cui Y., Yue Y., Qian G., Chen B. (2012). Luminescent Functional Metal−Organic Frameworks. Chem. Rev..

[31] Zhang H., Chen D., Ma H., Cheng P. (2015). Real-Time Detection of Traces of Benzaldehyde in Benzyl Alcohol as a Solvent by a Flexible Lanthanide Microporous Metal–Organic Framework. Chem. Eur. J..

[32] Cui Y., Chen B., Qian G. (2014). Lanthanide metal-organic frameworks for luminescent sensing and light-emitting applications. Coord. Chem. Rev..

[33] Xu L., Xu G., Chen Z. (2014). Recent advances in lanthanide luminescence with metal-organic chromophores as sensitizers. Coord. Chem. Rev..

[34] Roy S., Chakraborty A., Maji T. (2014). Lanthanide–organic frameworks for gas storage and as magneto-luminescent materials. Coord. Chem. Rev..

[35] Schubert E., Kim J. (2005). Solid-State Light Sources Getting Smart. Science.

[36] Higuchi T., Nakanotani H., Adachi C. (2015). High-Efficiency White Organic Light-Emitting Diodes Based on a Blue Thermally Activated Delayed Fluorescent Emitter Combined with Green and Red Fluorescent Emitters. Adv. Mater..

[37] Zhang X., Liu W., Wei G., Banerjee D., Hu Z., Li J. (2014). Systematic Approach in Designing Rare-Earth-Free Hybrid Semiconductor Phosphors for General Lighting Applications. J. Am. Chem. Soc..

[38] Zhang Y., Li X., Song S. (2013). White light emission based on a single component Sm(III) framework and a two component Eu(III)-doped Gd(III) framework constructed from 2,20-diphenyl dicarboxylate and 1H-imidazo[4,5-f][1,10]-phenanthroline. Chem. Commun..

[39] Su Y., Yu J., Li Y., Phua S., Liu G., Lim W., Yang X., Ganguly R., Dang C., Yang C., Zhao Y. (2018). Versatile bimetallic lanthanide metal-organic frameworks for tunable emission and efficient fluorescence sensing. Commun. Chem..

[40] Song Y., Duan F., Zhang S., Tian J., Zhang Z., Wang Z., Liu C., Xu W., Du M. (2017). Iron oxide@mesoporous carbon architectures derived from an Fe(II)-based metal organic framework for highly sensitive oxytetracycline determination. J. Mater. Chem. A.

[41] Zhang F., Yao H., Chu T., Zhang G., Wang Y., Yang Y. (2017). A Lanthanide MOF Thin-Film Fixed with Co_3_O_4_ Nano-Anchors as a Highly Efficient Luminescent Sensor for Nitrofuran Antibiotics. Chem. Eur. J..

[42] Zhao D., Liu X., Zhao Y., Wang P., Liu Y., Azam M., Al-Resayes S., Lu Y., Sun W. (2017). Luminescent Cd(II)–organic frameworks with chelating NH_2_ sites for selective detection of Fe(III) and antibiotics. J. Mater. Chem. A.

[43] Wang B., Lv X., Feng D., Xie L., Zhang J., Li M., Xie Y., Li J., Zhou H. (2016). Highly Stable Zr(IV)-Based Metal−Organic Frameworks for the Detection and Removal of Antibiotics and Organic Explosives in Water. J. Am. Chem. Soc..

[44] Wen L., Cheng P., Lin W. (2012). Mixed-motif interpenetration and cross-linking of high-connectivity networks led to robust and porous metal–organic frameworks with high gas uptake capacities. Chem. Sci..

[45] Binnemans K. (2015). Interpretation of europium(III) spectra. Coord. Chem. Rev..

[46] Eliseeva S., Bünzli J. (2010). Lanthanide luminescence for functional materials and bio-sciences. Chem. Soc. Rev..

[47] Haquin V., Etienne M., Daiguebonne C., Freslon S., Calvez G., Bernot K., Le Polles L., Ashbrook S., Mitchell M., Bünzli J. (2013). Color and Brightness Tuning in Heteronuclear Lanthanide Terephthalate Coordination Polymers. Eur. J. Inorg. Chem..

[48] Zhou Y., Yang Q., Zhang D., Gan N., Li Q., Cuan J. (2018). Detection and removal of antibiotic tetracycline in water with a highly stable luminescent MOF. Sens. Actuators B Chem..

[49] Yin K., Zhang W., Chen L. (2014). Pyoverdine secreted by Pseudomonas aeruginosa as a biological recognition element for the fluorescent detection of furazolidone. Biosens. Bioelectron..

[50] Zhang Z., Li M., Shen F., Ren X. (2015). Direct fluorescence quantification of sulfadiazine from quenching of novel functional monomer based molecularly imprinted polymers. Anal. Methods.

[51] Ma Y., Song Y., Ma Y., Wei F., Xu G., Cen Y., Shi M., Xu X., Hu Q. (2018). N-doped carbon dots as a fluorescent probe for the sensitive and facile detection of carbamazepine based on the inner filter effect. New J. Chem..

[52] Yang X., Liu M., Yin Y., Tang F., Xu H., Liao X. (2018). Green, Hydrothermal Synthesis of Fluorescent Carbon Nanodots from Gardenia, Enabling the Detection of Metronidazole in Pharmaceuticals and Rabbit Plasma. Sensors.

[53] Qin Z., Dong W., Zhao J., Wu Y., Tian Z., Zhang Q., Li D. (2018). Metathesis in Metal–Organic Gels (MOGs): A Facile Strategy to Construct Robust Fluorescent Ln-MOG Sensors for Antibiotics and Explosives. Eur. J. Inorg. Chem..

[54] Han M., Wen G., Dong W., Zhou Z., Wu Y., Zhao J., Li D., Ma L., Bu X. (2017). A heterometallic sodium–europium-cluster-based metal–organic framework as a versatile and water-stable chemosensor for antibiotics and explosives. J. Mater. Chem. C.

[55] Mulamattathil S., Bezuidenhout C., Mbewe M. (2015). Analysis of physico-chemical and bacteriological quality of drinking water in Mafikeng, South Africa. J. Water Health.

[56] Zhang X., Wang W., Hu Z., Wang G., Uvdal K. (2015). Coordination polymers for energy transfer: Preparations, properties, sensing applications, and perspectives. Coord. Chem. Rev..

[57] Wang X., Yao X., Huang Q., Li Y., An G., Li G. (2018). Triple-Wavelength-Region Luminescence Sensing Based on a Color-Tunable Emitting Lanthanide Metal Organic Framework. Anal. Chem..

[58] Banal J., Zhang B., Jones D., Ghiggino K., Wong W. (2017). Emissive Molecular Aggregates and Energy Migration in Luminescent Solar Concentrators. Acc. Chem. Res..

[59] Kulesza D., Bolek P., Bos A., Zych E. (2016). Lu_2_O_3_-based storage phosphors. An (in)harmonious family. Coord. Chem. Rev..

[60] Chen M., Yu H., Kershaw S., Xu H., Gupta S., Hetsch F., Rogach A., Zhao N. (2014). Fast, Air-Stable Infrared Photodetectors based on Spray-Deposited Aqueous HgTe Quantum Dots. Adv. Funct. Mater..

