# Supplementary material for: White-Light-Emitting Decoding Sensing for Eight Frequently-Used Antibiotics Based on a Lanthanide Metal-Organic Framework
Source: Polymers (Basel). 2019 Jan 9;11(1):99. doi: 10.3390/polym11010099 (PMC6402005; doi:10.3390/polym11010099)
Supplement: Supplementary file 1 [file polymers-11-00099-s001.pdf]

Supporting Information

White-light-emitting decoding sensing for eight frequently-used antibiotics based on a lanthanide metal-organic framework

Mingke Yu, Yuxin Li*, Guangming Li*

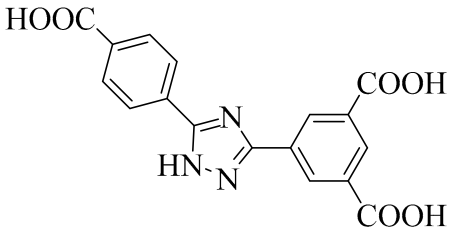

Scheme S1. The structure of the ligand H_3dcpcpt.

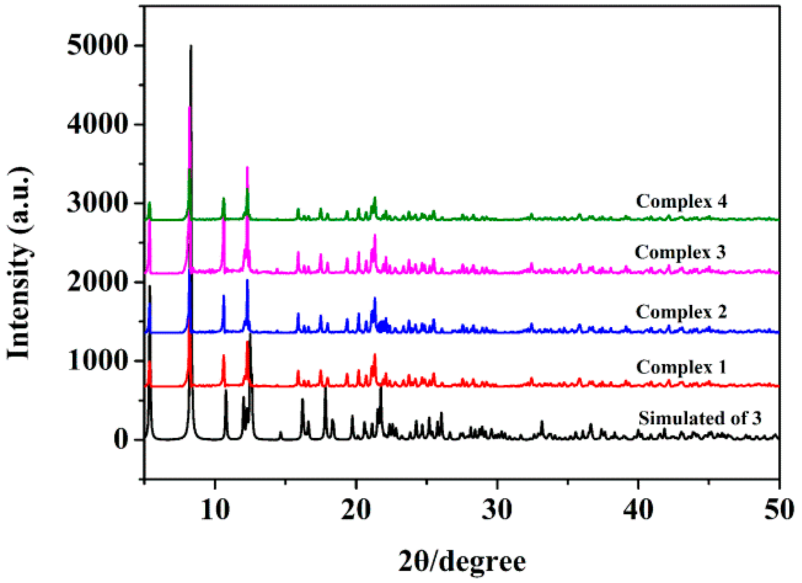

Figure S1. PXRD patterns of complex **3** simulated from the X-ray single-crystal structure and as-synthesized samples of complexes **1**–**4**.

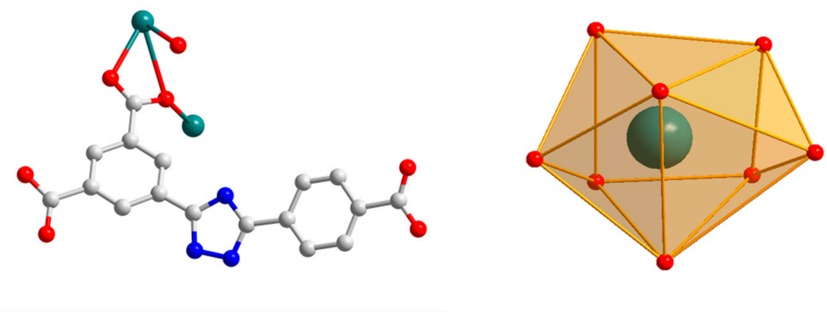

Figure S2. (a) The asymmetric unit of complex **3**. All hydrogen atoms and solvent molecules are omitted. (b) Coordination polyhedrons of Tb^{3+} ion.

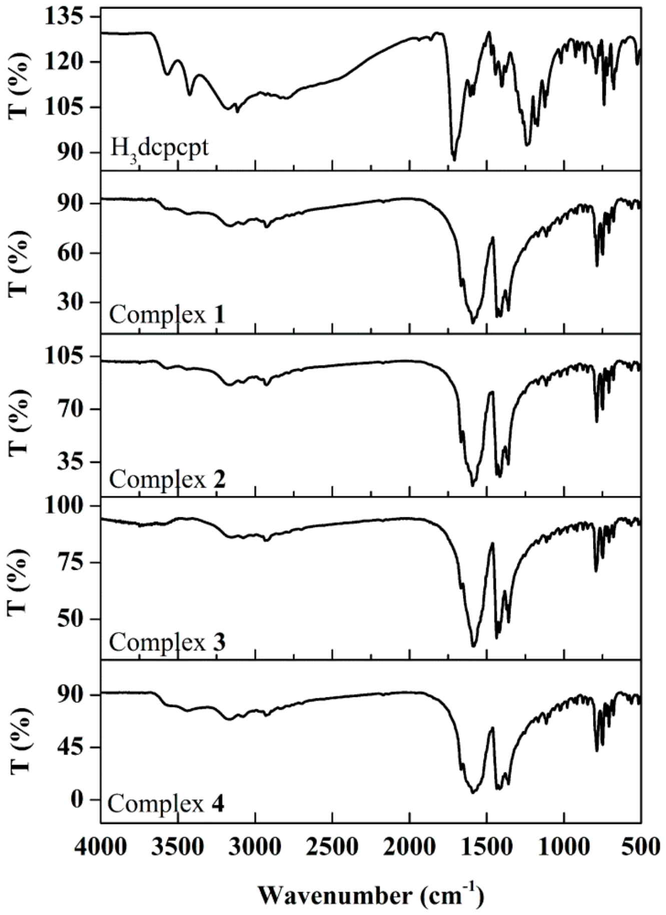

Figure S3. FT-IR spectra of ligand and complexes **1**–**4**.

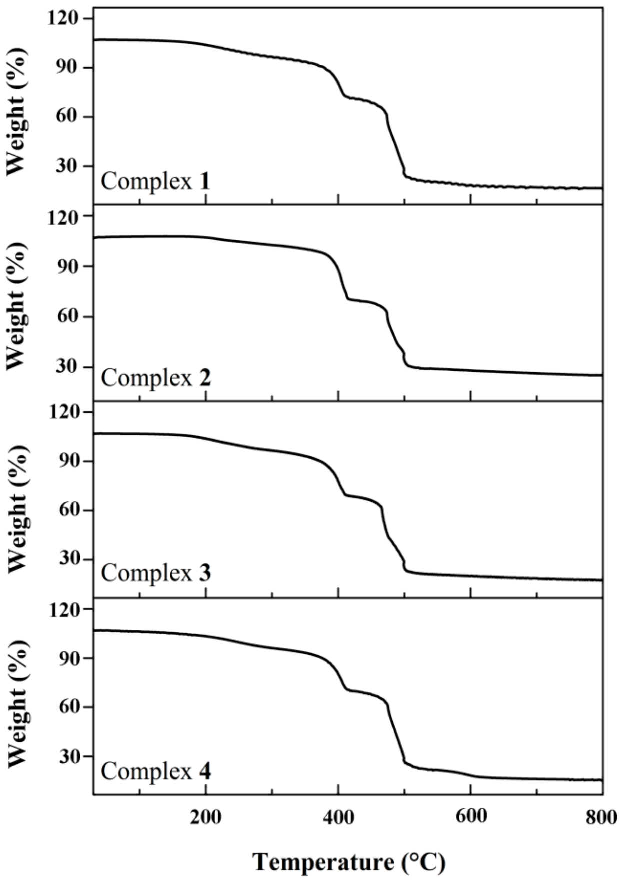

Figure S4. TG curves of complexes **1**–**4.**

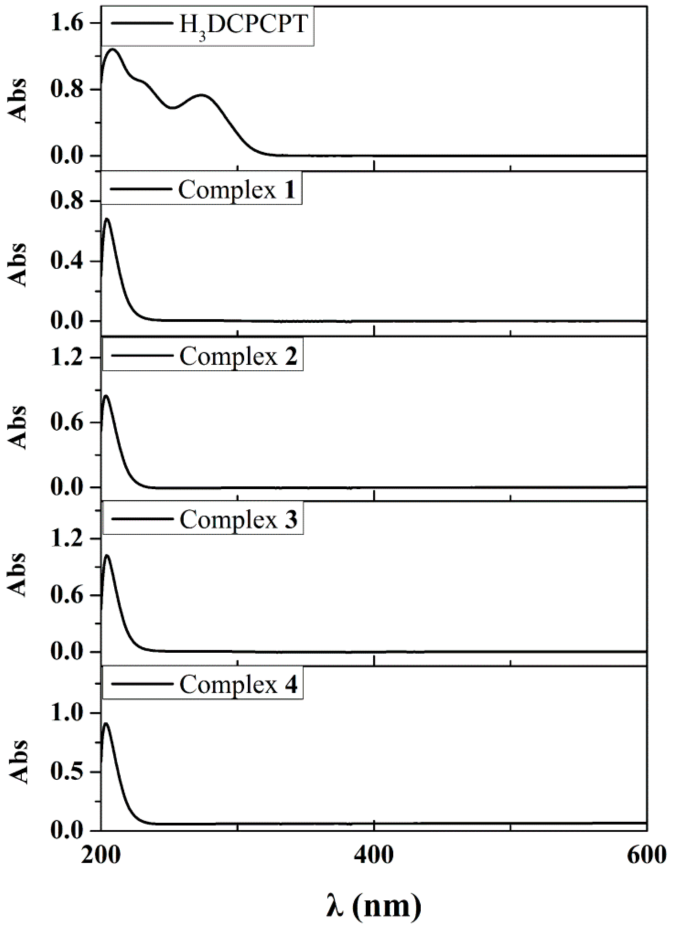

Figure S5. UV-vis spectra of ligand and complexes **1–4.**

Table S1. Elemental analysis of lanthanide ions by ICP for complex **4**.

Sample	Complex **4**		
	Eu	Gd	Tb
Starting Eu, Gd and Tb salt (%)	40	40	20
The ratio by ICP analysis (%)	41	42	17

Table S2. Luminescence lifetime of complexes **1**–**4**.

No.	τ (ms)		
	@439 nm	@546 nm	@615 nm
**1**	-	-	0.32
**2**	3.2×10^{-3}	-	-
**3**	-	1.24	-
**4**	8.4×10^{-4}	0.28	0.24

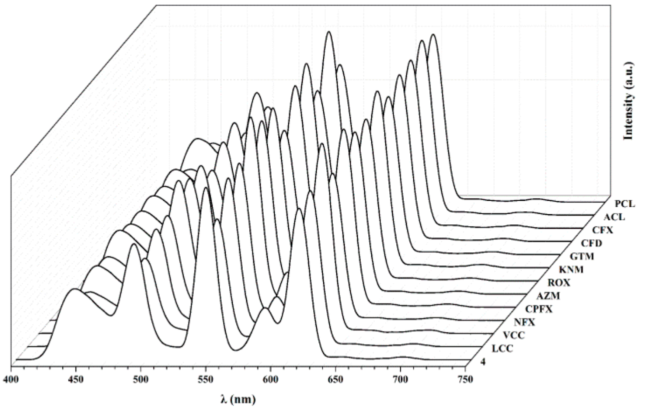

Figure S6. Emission spectra of antibiotics@**4** excited at 320 nm.

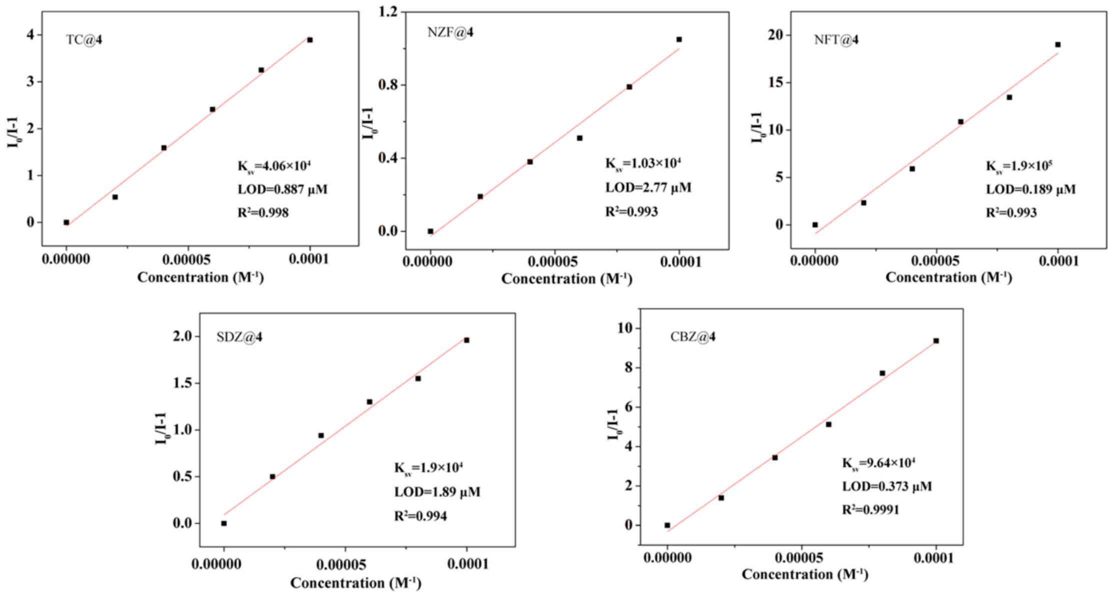

Figure S7. Stern-Volmer plot of TC@**4**, NZF@**4**, NFT@**4**, SDZ@**4** and CBZ@**4**antibiotics@**4**.

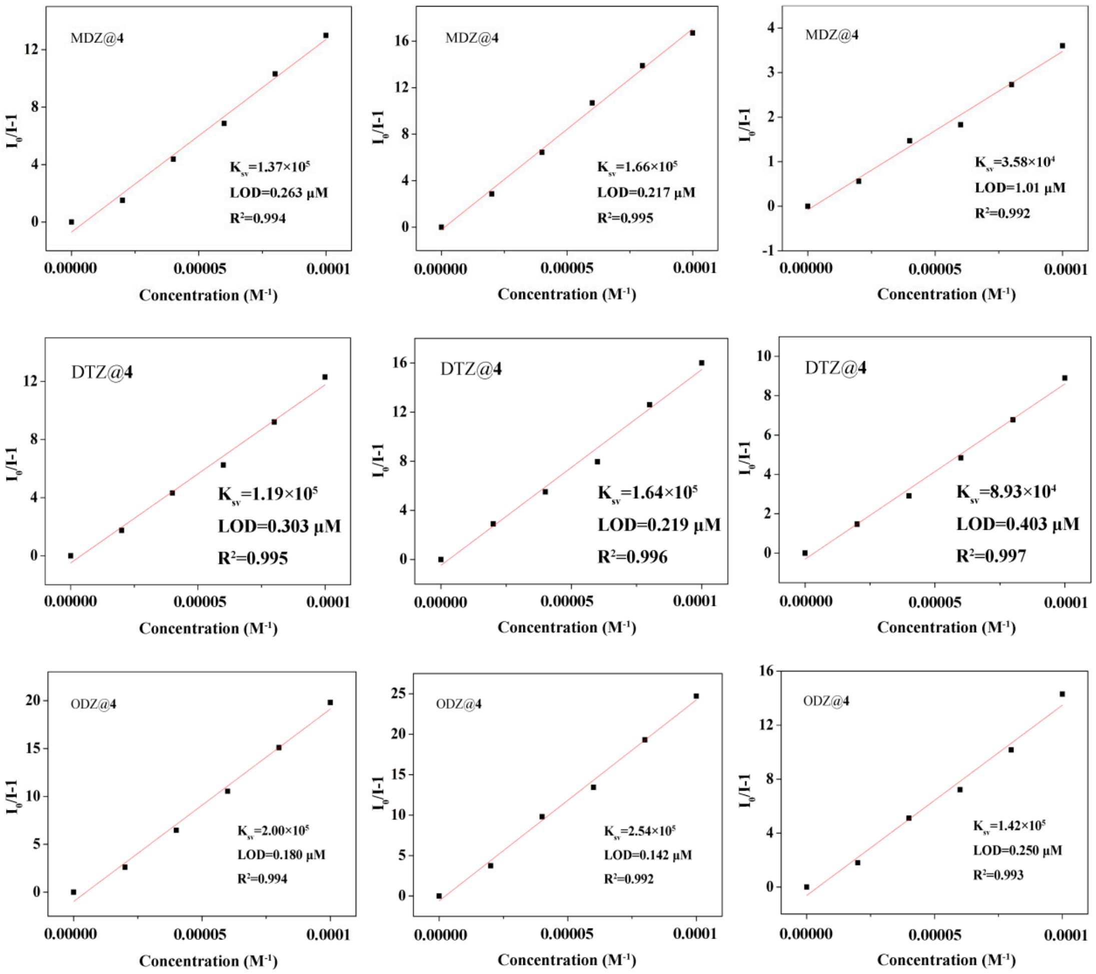

Figure S8. Stern-Volmer plot of MDZ@**4**, DTZ@**4** and ODZ@**4**.

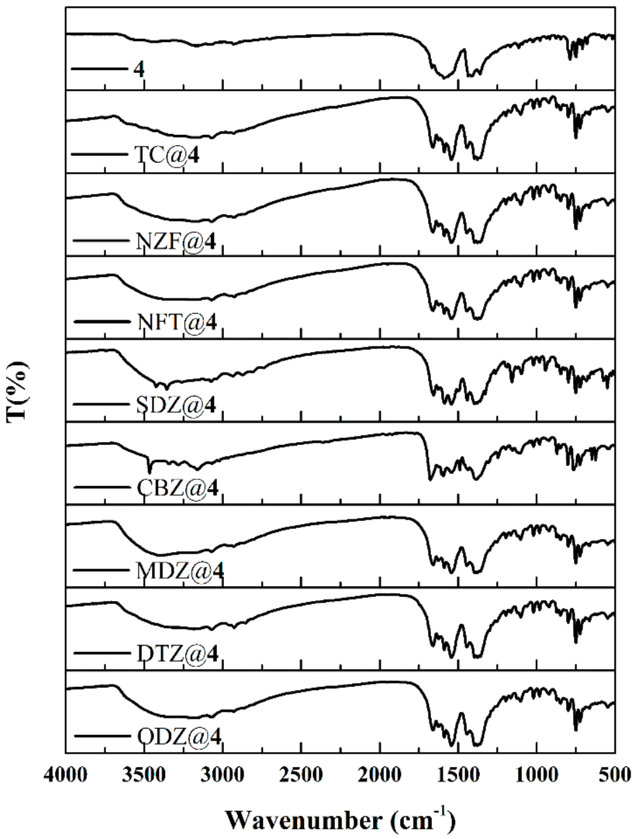

Figure S9. FT-IR patterns of complex **4** before and after sensing antibiotics.

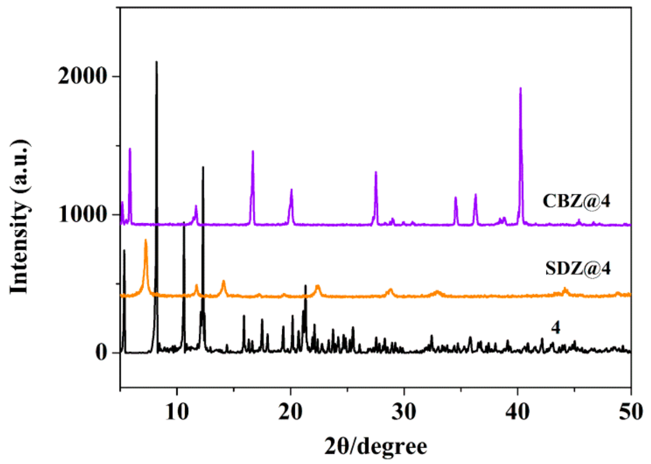

Figure S10. PXRD patterns of complex **4** before and after sensing SDZ and CBZ.

Table S3. Peak analysis for **4**.

Wavenumber (nm)	Height	FWHM	Area
439.2	554.7	21.3	630.1
458.7	640.0	31.7	1082.7
490.9	1328.4	20.1	1421.8
515.7	149.8	19.5	155.6
545.1	2038.8	17.8	1926.9
590.6	586.3	21.6	672.9
613.8	1802.1	17.2	1653.5

Table S4. Peak analysis for TC@**4**.

Wavenumber (nm)	Height	FWHM	Area
450.1	458.4	12.2	651.3
471.6	322.4	15.8	569.7
493.6	317.8	12.2	391.5
523.9	331.4	108.9	1423.1
545.0	300.8	16.5	321.7
591.9	202.0	7.9	221.5
614.5	1546.0	17.2	1167.1
